# Playing the Admission Game: Young People and Their Parents Negotiating Access or Discharge to Adolescent Inpatient Mental Health Services

**DOI:** 10.1111/jcap.70006

**Published:** 2024-12-10

**Authors:** Anna Sydor, Rhiannon Lane, Nicola Evans

**Affiliations:** ^1^ Department of Health care sciences Cardiff University Cardiff Wales UK; ^2^ Department of Sociology Exeter University Exeter UK

**Keywords:** admission, discharge, inpatient, mental health, young person

## Abstract

**Background:**

Only children and young people with the highest need for mental health care or support are admitted to an inpatient setting. There has been a recent shift in emphasis with the aim of inpatient care being short and focussed, care is transferred back to the community on discharge. Little is known about what young people and their parents understand about admission and discharge criteria to these inpatient facilities.

**Purpose:**

This exploratory study aimed to explore the perspectives of young people (aged 18–25) and parents of young people regarding the reasons for their past admissions (or nonadmission) to inpatient child and adolescent mental health services (CAMHS), including beliefs concerning the reasons for subsequent discharge.

**Methodology:**

Data were conducted in various ways according to participant preference either in person telephone or written interview. Participants were young people (*n* = 5) or parents of young people (*n* = 5). Thematic analysis was used to identify emerging themes collaboratively.

**Results:**

Based on the three themes that were identified: power control and choice, seeking knowledge and taking control, and conflicting notions of recovery and health we found that young people and their patents were engaged in a complex interaction in which they played the admission game; negotiating admission or discharge through behaviors and counterbalancing risks and benefits.

**Conclusion:**

Understanding the complexity of this interaction may help professionals, including nurses to support patients and their families during the admission, care planning or discharge process and to recognize risks to prevent them escalating.

## 1 Background

In the United Kingdom and internationally, levels of mental health problems in adolescents and young people (YP) have been rising, with recent estimates suggesting that one in five children (17.4%) aged 6–19 have children a diagnosable mental health problem in the United Kingdom (NHS England 2023). Internationally, 8.8% of children have been diagnosed with a mental illness (Piao et al. 2022) and many more experience symptoms without diagnosis. There have been increasing demands for care and support, with services struggling to meet the increasing needs of YP and their families (Department of Health 2015; National Assembly for Wales Children Young People and Education Committee 2014).

Children and adolescents with the highest levels of need are generally cared for within inpatient settings, which is thought appropriate due to the 24‐h care provided, better ensuring the safety of YP who may be deemed at risk of harm. However, there is a high demand for beds and a general lack of agreement regarding the criteria for admission to, and discharge from, such units (Evans, Edwards, and Carrier 2020). Decisions on who to admit to inpatient child and adolescent mental health services (CAMHS) in the United Kingdom often occur within the context of limited bed capacity, and largely revolve around perceptions of risk, although this can vary upon external triggering factors and context. Perhaps due to this increase in demand for mental health care combined with the shortage of inpatient capacity, research has shown that negotiating access to inpatient beds for adolescents can be challenging (Stanton, Lahdenperä, and Braun 2017).

Focus and function of inpatient care has changed as effective community‐based interventions for common mental health presentations in adolescents have been developed (Lamb 2009). In CAMHS—as with mental health care generally—there has been an increasing emphasis upon shorter admissions and treatment within the community (Blanz and Schmidt 2000; Fennig, Fennig, and Roe 2002; Henggeler et al. 1999) (despite evidence that longer stays are associated with better outcomes, e.g, Green et al. 2007). However, it is not certain whether this preference for community treatment is shared by YP seeking care for mental health difficulties.

A scoping review (Evans, Edwards, and Carrier 2020) found a lack of research exploring the perspectives of adolescents and their families or carers about what constituted criteria warranting admission to inpatient mental health care, indicating an important area for future investigation. Research on professional beliefs and knowledge regarding admissions criteria was found (Stanton, Lahdenperä, and Braun 2017); however, it is important to consider the nature of patient (and carer) beliefs regarding inpatient admission criteria, and how they might compare with the views of professionals who run services. Such beliefs are likely to influence the likelihood of a young person presenting or being presented to inpatient or crisis services; secondly, beliefs regarding admissions criteria are likely to shape the way in which YP (and their carers) interact with services and professionals, respond to care and treatment, respond to being admitted to inpatient care, and engage with post‐discharge aftercare.

### 1.1 Aims

This is a qualitative descriptive study; it lays a foundation for further research (Kothari 2017).

The aim of the study was to explore YP's (aged 18–25) and their parents' beliefs regarding the reasons for their past admission/s (or nonadmission) to inpatient mental health facilities, including beliefs regarding the reasons for subsequent discharge.

## 2 Methods

This qualitative descriptive study (Doyle et al. 2020) aimed to explore how participants make sense of their own admissions or non‐admissions and discharges from inpatient services, making it vital to elicit their own accounts and understandings of this. The study was underpinned by a social constructivist approach, individuals live in their own reality and understand their experiences in light of this (Boyland 2019) but understandings of mental health and illness are also constructed socially and are located within culture, time and place (Burr 2015; White 2017).

Ethical approval was granted by the Cardiff University ethics committee.

### 2.1 Participants

We recruited participants through Facebook groups created by patients or their families to discuss their care by CAMHS. The lead researcher (R.L.) requested membership of these groups, stating that a research study was being undertaken and participants were sought. The lead researcher posted in the group with the group administrators' approval, potential participants responded to this post and were provided with a participant information sheet and allowed time to consider taking part. Potential participants were given a choice about how they wished to participate. Participants were parents of patients admitted to CAMHS (*n* = 5) and YP (*n* = 5) who had been admitted to a CAMHS unit. Some responses (*n* = 2) were from YP who had received mental health care but not as an inpatient, this did not become apparent until after interviews had commenced. These data were included in the analysis as it illuminated the complexities surrounding mental health care. YP were aged 18–25 and parents of YP of the same age, this was so that they would have had opportunity to reflect following discharge or care incidence.

### 2.2 Interviews

The primary researcher (R.L.) undertook all data collection. This was a face‐to‐face or telephone interview or a series of email questions. Face‐to‐face and telephone interviews yielded the richest data but many YP preferred to answer written questions. Data included face‐to‐face interviews, telephone interviews and email interviews (see Table 1). Such computer‐mediated methods enabled the research to include isolated, geographically dispersed and stigmatized groups who may be overlooked (McCoyd and Kerson 2006). Participation in groups enabled prolonged engagement with the culture of participants (Johnson, Adkins, and Chauvin 2020), two researchers remained outside the groups to regulate bias and facilitate reflexivity in discussion of findings (Yadav 2022).

**Table 1:** Presence of themes in participants narratives.

Participant	Interview type	Theme: Power, control and choice	Theme: seeking knowledge and taking control	Theme: Conflicting notions of recovery and health
Parents 1 and 2: Parents of a child who was admitted to a general hospital ward and then a CAMHS inpatient unit	Face to face	P	P	P
Parent 3 of a child who was admitted to CAMHS units on three occasions	Telephone interview	P	P	P
Parent 4 of young person accepted into CAMHS services	Email interview	P	P	
Parent 5 of young person admitted to CAMHS unit	In‐person interview	P	P	P
Young person 1 admitted to CAMHS unit	Email interview	P	P	x
Young person 2 admitted to both NHS and private CAMHS units on separate occasions	Telephone interview	P	P	P
Young person 3 admitted to CAMHS unit on one occasion	Email interview	P		P
Young person 4 admitted to hospital ward for psychiatric symptoms	Email interview		P	
Young person 5 treated under CAMHS, admitted to hospital following self‐harm, and attended psychiatric hospital as an outpatient	Telephone interview		P	

### 2.3 Analysis

Data were analyzed using thematic analysis (Braun and Clarke 2021). The procedure followed the six‐stage approach. Interviews were transcribed by two researchers (R.L., A.S.) and then imported into NVivo v12, which assisted with data management and communication of themes. Data were analyzed by all authors who took part in comparative discussions. Analysis was iterative and involved returning to transcripts repeatedly; transcripts and coding structures were considered by all authors. Participant checking of themes was not offered owing to the sensitive nature of the topic under consideration and recognition of the constructivist and interpretivist stance that we adopted (Varpio et al. 2017).

## 3 Findings

Three superordinate themes were generated from the data (Braun and Clarke 2019), these themes were evident in data collected from both YP and parents. Participants have been numbered and reference to patient characteristics removed to preserve confidentiality.

1. **Lack of power, control and choice**.
  Parents and YP were concerned about lack of choice and control over care offered. This theme was present in their discussion of all aspects of care from the point of presentation to admission and discharge.
  Both parents and YP voiced distress about the admission process; despite this having been different for all participants. Parents 1 and 2 contrasted the care received in a mental health facility with that provided on a general children's ward using the disparity to explain why they felt negative about the mental health facility:
  and I don't know if you are spoiled in the children's ward….
  (Parent 1)
  YP 1 felt that they had no control over the care they received and spoke about the care received from different professionals as being quite different:
  I guess interaction with police I've had—so I've been sectioned under 136 section—emm and that was at a station. And the rest of the time when police had to come—they're often much more caring than mental health professionals. They actually listen to you
  (Young person 1)
  This variation left a feeling of lack of control, those delivering the care for YP have an impact on the experience, but the young person has little control over who these people will be or how they will be treated. This contrast was also evident in emergency unit care:
  A & E [accident and emergency unit] staff again are way better than mental health professionals because they listen to you … like you don't necessarily want expertise, you just want someone to see that you're desperate really
  (Young person 1)
  YP and parents described sectioning being used as a tool if they did not feel the admission or care was appropriate; meaning that their choices could always be overruled.
  Both parents and YP voiced concerns over lack of choice and control; whether they were admitted or not, whether or when they were discharged and the place or nature of their admission. Access to appropriate care often involved narratives of chance encounters and luck. For YP, these narratives tended to imply a lack of active help seeking and an identification of risk from important others (e.g., teachers, counselors, or parents).
  For parents, this reflected a lack of knowledge of the pathways to appropriate help, making both luck and advice from others important in obtaining access to higher levels of care for their children. Care was not ideal for families, but participants expressed awareness that it could be much worse.
2. **Seeking knowledge and taking control. **
  Most of those interviewed were familiar with psychiatric terminologies and systems. The issue of diagnosis came up frequently, without prompting. There was generally an awareness surrounding diagnostic terminology and an eagerness to obtain a diagnostic label. This desire for diagnosis appears to be partly a means to self‐understanding but also functioned to legitimize the YPs difficulties through medicalisation.
  Parents interviewed fought for appropriate care for their children, taking on the role of advocates, familiarizing themselves with policies and legal concepts surrounding the provision of mental health care. Parents sometimes referred to their role as pivotal:
  So, how I got my son in an area was…
  (Parent 3)
  However, despite the obvious benefits of a proactive approach parents or YP may be perceived to be difficult or overly demanding. For example, Parents 1 and 2 reported that asking for a different support worker for their daughter led to no support worker, they were told:
  we'll listen to what you are saying you know, we'll give you another worker but you know…we haven't got anybody at the moment
  (Parent 1)
  YP felt that asking for help rendered it less likely to be provided, as this demonstrated insight into their condition. They discussed ways that they covertly but proactively sought continued admission:
  I think the only way you can stay in hospital is to make them think you don't want to be in hospital because they see that as an indication of risk and unwellness ‐ we would routinely do things like get ourselves forcibly medicated and restrained and sectioned and… even though those are hugely traumatic things—that was the only way of them taking you seriously
  (Young person 2)
  In contrast Young person 1 described being sectioned during an inpatient stay, due to demanding to leave, Young person 3 described a form of informal sectioning, or detention:
  I'm not sectioning you now but if you try to leave, we will’ so I was basically detained
  (Young person 3)
  This seems to depict a situation where those who do not want to be admitted are more likely to be admitted, while those seeking admission are less likely to.
3. **Conflicting notions of risk, recovery and health.**
  There were conflicting notions of wellness between professionals and parents/YP. Within inpatient settings the focus for professionals tended to be described as the physical aspects of wellbeing (e.g., weight, outward physical appearance) and physical risk (risk of suicide or physical self‐harm) meaning that both parents and YP described having emotional experiences ignored.
  A focus on the physical display of distress could also lead to the need to exhibit distress in a visual way to be validated and receive support. This meant it was not sufficient to simply ask for help or to report feelings of extreme distress, there was a perceived need to outwardly display these feelings so that they could be seen by others. Consistent with this, few of the YP indicated having *explicitly* asked for help, this was depicted as having been initiated by others (e.g., teachers, parents, counselors) who had noticed visual clues about the YP's mental state. Admission was described as an emergency intervention but not considered as a beneficial long‐term treatment.

## 4 Playing the Admission Game

**Figure 1:**
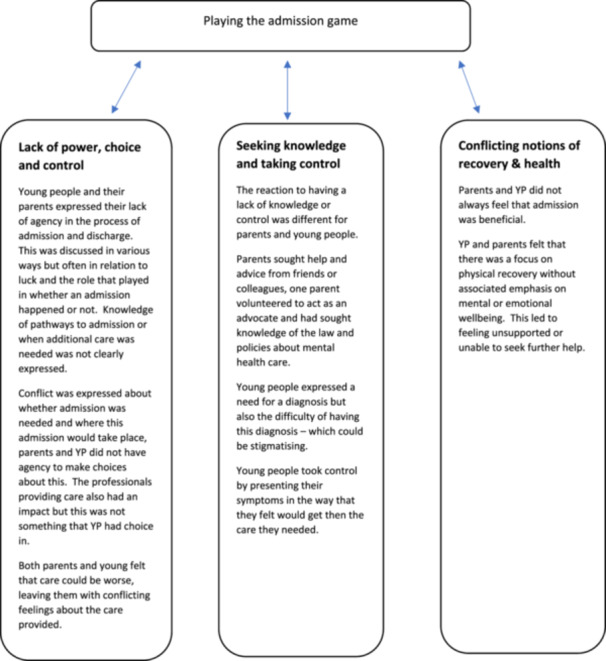
Summary of themes. [Color figure can be viewed at wileyonlinelibrary.com]

The complex interplay between negotiating admission and discharge, maintaining safety and managing risk, and maintaining choices were named playing the admission game—this idea explains the participants discourses set out in the themes identified (Figure 1).

YP discussed ways that they had either perpetuated an existing admission or gained admission to a mental health facility when they felt it was required, the discourse of both YP and their parents suggested that they did not feel emotional or psychological distress would be perceived sufficient reason to be admitted. Instead, physical symptoms were focused on for example weight loss or self‐harm. One YP explained choosing to exhibit behaviors to remain an inpatient. This contrasted with situations in which there was conflict about care between parents or YP and health care professionals, forced treatment (being detained under the Mental Health Act 2007) was threatened if care plans were not agreed. Being admitted to the hospital either voluntarily or being detained under the Mental Health Act (Her Majesty's Government 2007) was experienced as disempowering for YP, whose admission was brokered by others, professionals or parents, not instigated by their own enquiry. An understanding of this interaction is important for nurses and health care professionals providing information and supporting care plans for YP. Discourse of information provision impacted the YP and their parents and provided opportunity to include them in care planning or leave them feeling disempowered (Viksveen et al. 2022).

Both YP and their parents were unclear about admission and discharge criteria; feeling that admission only provided an opportunity to keep a YP safe in emergencies and focused on physical symptoms and needs, rather than the underlying mental health issues. Parents did not feel that significant improvement was made with these underlying issues before discharge and so the admission was seen as addressing only physical symptoms. Nurses have an opportunity to share information about admission and discharge requirements, both to enhance empowerment but also to provide reassurance about the focus of an admission.

While parents focused on the physical benefits of admission, namely weight gain or prevention of physical injury both YP and parents felt the need to be cared for. YP spoke about mental health professionals being uncaring in comparison to police and parents contrasted the mental health inpatient care with general care that they felt had “spoiled” them. Thus, admission to a mental health inpatient facility became a risk only worthwhile if benefits could be gained; furthermore, when admission had taken place both parents and YP felt some lack of control and reduced ability to make decisions about their care. Parents described a lack of communication and no input in decisions about the care of their child. One parent described their child going missing from an inpatient unit, a situation only shared with them after its resolution. In this the role of nurses is central, it is important that speaking with care and empathy is prioritized.

Provision of inpatient mental health care is nuanced and complex, the nature of mental health diagnoses and illness is not fixed (Jerotic et al. 2024) nor can it be viewed outside a wider societal lens. Participants alluded to this when considering admission and discharge which was a situation that could be influenced. Parents spoke about this as taking control and used resources available to them to manage navigating the system and obtain the care that they felt was needed. Resources were individual but included colleagues and voluntary roles. The nature of these resources meant that socioeconomic status impacted a parent's access to them (Currie, Kurdyak, and Zhang 2024; Rice et al. 2018), socioeconomic background may impact diagnosis (Kirkbride et al. 2024). This means that the ability of the parents and YP to access inpatient services and exercise agency may be contingent on their wider societal position. One YP spoke about this explicitly when considering the different between public and private health care. A feeling of helplessness has been identified by parents of children with mental health diagnoses, Seney (2024) also found that parents did not know where to access help or how to navigate the care system.

The issue of being given a diagnosis was complex; if it were an “acceptable” diagnosis it was validating but diagnosis did not necessarily lead to different care being offered. There was a conflict between a need for a diagnosis and care and a loss of power and control when care was made available. This was often because it was not provided in a way that the young person or their parent found acceptable, far away from home or the parent felt insufficiently involved and communicated with.

The admission game discussed here is a complex interaction in which parents and YP seek to gain mental health care, often in crisis situations. The possible risks of doing this are great for both parents, who felt loss of control and YP who felt uncared for or had to accept care a long way from home. Any subversion from the prescribed care could lead to it being enforced, a situation which parents and YP wished to avoid. The need for YP to be kept safe was paramount to parents, whose discourse was one of concern. The activities they undertook to take control reflected this concern, although not all parents had access to the same resources to support them with this.

### 4.1 Strengths and Limitations

Findings of this study must be considered within a wider context. Participants were recruited through online support groups, the membership of which was largely comprised of those who felt negatively about the mental health care, it is not known how those parents or YP who had a positive experience of care would describe it. The study was also limited by the varying methods of data collection. Written answers were not as comprehensive as data gained from interviews, but for some participants this was the only way that they were willing to offer their experiences. The retrospective nature of the study may have influenced the way events were recalled or understood but was chosen to safeguard potentially vulnerable participants.

The study offers insight into these experiences, which facilitate an understanding into the wider phenomenon and may help health care professionals to understand patients and their families.

A framework to assure quality was used (Yardley 2000), this informed both our approach to the research question and sensitivity to approaching the discussion with potential participants as well as the practical and theoretical nature of findings, which seek to inform nursing practice.

### 4.2 Implications for Practice

YP require inpatient mental health services when their mental health is significantly impaired. It is only provided in the UK at times when community services would not be able to offer sufficient care. This study has highlighted that YP and their families feel disempowered due to the power differences; health and social care practitioners need to be aware of this power differential when admitting or discharging YP.

The reasons for admission are not always understood. It might be useful for these to be clearly articulated, captured in a care plan in a written format and explained as the young person requires. Where practicable, parents should be involved in decision making about their children, despite inpatient units being geographical distant parents should be engaged at critical points; remote facilities such as Zoom or Teams could be a useful medium.

### 4.3 Implications for Further Research

This was a small exploratory study which began the investigation of the experiences of YP and families of inpatient mental health care. This captures the views of a small number of people recruited through social media but does illuminate the serious issues of power imbalance and control in mental health admissions and discharges. It would be useful to undertake further studies to reveal the complex dynamics that occur, from which candidate interventions to address such unwanted experiences might be developed.

## Author Contributions

**Anna Sydor:** data analysis, article preparation. **Rhiannon Lane:** data collection, analysis, article preparation. **Nicola Evans:** conceptualized project, article preparation.

## Conflicts of Interest

The authors declare no conflicts of interest.

## Data Availability

Research data are not shared, this is due to the likelihood of participants being identified from the full data—numbers of children admitted to mental health units are not large and experiences are described in detail. To protect participants anonymity full data (interview transcripts) will not be made available.
